# Dietary Mineral Intake and Risk of Mild Cognitive Impairment: The PATH through Life Project

**DOI:** 10.3389/fnagi.2014.00004

**Published:** 2014-02-04

**Authors:** Nicolas Cherbuin, Rajeev Kumar, Perminder S. Sachdev, Kaarin J. Anstey

**Affiliations:** ^1^Centre for Research on Ageing, Health and Wellbeing, Australian National University, Canberra, ACT, Australia; ^2^Medical School, Australian National University, Canberra, ACT, Australia; ^3^Neuropsychiatric Institute, University of New South Wales, Sydney, NSW, Australia

**Keywords:** magnesium, potassium, calcium, iron, dementia

## Abstract

**Background:** Higher dietary intake of potassium, calcium, and magnesium is protective against ischemic strokes while also being associated with a decreased risk of all-cause dementia. The effect of dietary iron intake on cerebral function is less clear but iron is also implicated in Alzheimer neuropathology. The aim of this study was to investigate whether dietary intake of these minerals was also associated with increased risk of mild cognitive impairment (MCI, amnestic) and other mild cognitive disorders (MCD).

**Methods:** Associations between dietary mineral intake and risk of MCI/MCD were assessed in cognitively healthy individuals (*n* = 1406, 52% female, mean age 62.5 years) living in the community, who were followed up over 8 years. Relative risk was assessed with Cox hazard ratios (HRs) after controlling for health and socio-demographic covariates.

**Results:** Higher magnesium intake was associated with a reduced risk of developing MCI/MCD (MCI: HR 0.07, 95% confidence interval (CI) 0.01–0.56, *p* = 0.013; MCD: HR 0.47, 95% CI 0.22–0.99, *p* = 0.046) in multivariate analyses. Higher intake of potassium (MCI: HR 1.09, 95% CI 1.01–1.17, *p* = 0.028; MCD: HR 1.05, 95% CI 0.99–1.10, *p* = 0.107) and iron (MCI: HR 1.54, 95% CI 1.03–2.29, *p* = 0.034) was associated with an increased risk of developing MCI/MCD.

**Conclusion:** These findings suggest that dietary intake of minerals known to be implicated in biological processes associated with vascular and Alzheimer’s pathology may contribute to disease progression earlier in the disease process and require further attention.

## Introduction

Worldwide dementia prevalence is projected to increase substantially in the coming decades. In addition to lowering the quality of life of sufferers and their family, this disease will put significant strain on health and social services as well as incurring important economic costs. As no cure or treatment for dementia is currently available it is critical that, where possible, preventative actions be implemented. Emerging evidence suggests that some dietary minerals (calcium, magnesium, potassium) are associated with lower dementia risk. Since diet is highly modifiable and exerts its influence over the lifespan, it is imperative that we better understand the associations between dementia and mineral intake, particularly in the early stages of the disease where interventions are most likely to be effective.

In a study of more than 1000 older individuals with a 17-year follow-up Ozawa et al. (2012) showed that higher intake of calcium, magnesium, and potassium was associated with a lower risk of developing all-cause dementia, particularly vascular dementia. In other studies, consistent protective effects of these minerals were found in relation to hypertension and stroke (Iso et al., 1999; Houston and Harper, 2008; Larsson et al., 2008). Higher magnesium and calcium plasma levels were also found to be associated with a lower risk of insulin resistance and type 2 diabetes (Larsson and Wolk, 2007; Villegas et al., 2009; Wu et al., 2009), which are known risk factors for dementia and poorer cognition (Cheng et al., 2012). Moreover, individuals suffering from Alzheimer’s disease (AD) have been shown to have lower plasma magnesium levels (Barbagallo et al., 2011). Together, this evidence suggests a consistent link between intake of calcium, magnesium, potassium, and future risk of cognitive decline.

Iron is another mineral thought to be implicated in Alzheimer neuropathology. It has been found to co-localize with amyloid plaques (Beauchemin and Kisilevsky, 1998; Dong et al., 2003) and is thought to be implicated in the pathological process leading to amyloid plaque aggregation (Roberts et al., 2012). Iron plasma levels have also been found to be positively associated with cognitive function in elderly individuals (Yavuz et al., 2012). These associations were detected even in the absence of anemia, where low iron levels might lead to lower hemoglobin levels and possible cerebral hypoxia, consequently other mechanisms might also be at play.

It is unclear, however, when associations between dietary minerals cognitive impairment become detectable in the prodromal stages of dementia, the extent to which these associations are due to interactions between different minerals, and whether gender differences exist. In the present research, we investigate associations between dietary mineral intake and the development of prospective mild cognitive disorders, including mild cognitive impairment (MCI) while testing mineral–mineral and sex interactions. A particular strength of this study is that we focus our analyses on a large prospective cohort (*n* = 2551) of individuals who were cognitively unimpaired at the first assessment, live in the community and who are taking part in the PATH through life project. The PATH project is a large longitudinal study of aging and is unique in that it randomly samples individuals in their early 60s from the population, uses a narrow age-range design which minimizes aging and cohort effects, and re-assesses participants every 4 years over a very broad range of socio-demographic, health, lifestyle, and neuropsychological measures. Based on research reviewed above, we predicted that higher dietary intake of calcium, magnesium, potassium, and iron would be associated with a decreased risk of progressing from normal cognition to MCI and other mild cognitive disorders over an 8-year follow-up.

## Materials and Methods

### Study population

**Figure 1:**
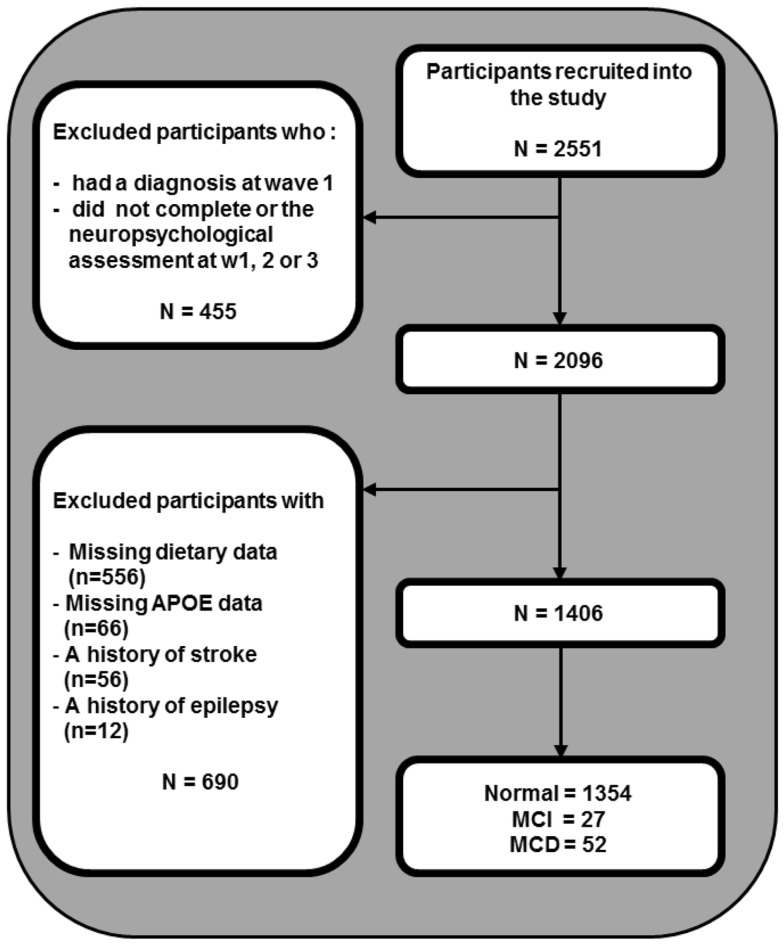
**Sample selection**.

The design of the PATH through life study has been described elsewhere (Kumar et al., 2005). Briefly, we recruited participants who were residents of the city of Canberra and the adjacent town of Queanbeyan, Australia, randomly through the electoral roll to participate in a study interested in the risk and protective factors for common mental disorders, normal aging, and dementia. Enrollment to vote is compulsory for Australian citizens. The Australian National University Ethics Committee approved the study and all participants gave written informed consent to be included in this study and the participation rate was 58.3% (2551 out of 4376 participants invited). The present investigation focuses on the middle-aged cohort, aged 60–64 years at wave 1 (2001–2002), 65–69 years at wave 2 (2005–2006), and 69–72 years at wave 3 (2009–2010). From 2551 participants recruited into the study, 455 were excluded because they either had a clinical diagnosis at wave 1 or did not complete the neuropsychological assessment at any assessment, 556 individuals were excluded due to missing dietary data at first assessment, 66 due to missing APOE data, 56 for stroke, and a further 12 for epilepsy leaving 1406 participants for analysis (Figure 1). Compared to 877 individuals who were not considered due to missing data, the selected participants did not differ in age but had higher levels of education (13.98 vs. 13.34 years; *t*(2549) = −5.277, *p* < 0.01) and were more likely to be women (51.1 vs. 43.1%; Chi-square = 14.872, df = 1, *p* < 0.01).

### Screening

At each wave, the same predetermined cutoff on a cognitive screening battery was used to screen participants into a substudy on MCDs and dementia. Participants from the full cohort were selected for clinical assessment if they had any of the following: (1) a Mini-Mental State Examination score ≤25; (2) a score below the fifth percentile on immediate or delayed recall of the first trial of the California Verbal Learning Test (immediate or delayed score of <4 and <2, respectively); (3) a score below the fifth percentile on either of the following two tests: Symbol-Digit Modalities Test (<33) or Purdue Pegboard with both hands (wave 1: <8; wave 2: <7) or reaction time (third set of 20 trials) (wave 1: >310 ms; wave 2: >378 ms).

### Clinical assessment

The clinical assessment involved a Structured Clinical Assessment for Dementia (available from us) by one of two physicians, a neuropsychological assessment, and the Clinical Dementia Rating Scale (Morris, 1993). Information was also gathered on medical history related to cognitive function, duration of symptoms, medical history from medical practitioners and family, current treatment, and psychiatric history. Depression was assessed using the self-administered Patient Health Questionnaire from the Primary Care Evaluation of Mental Disorders (PRIME-MD) (Spitzer et al., 1999). Informant interviews were conducted when possible (57% of participants with MCI). Participants receiving any clinical diagnosis were referred to their family doctor for laboratory investigations. When necessary for diagnostic purposes the research protocol included magnetic resonance imaging for all consenting participants. The neuropsychological assessment included frontal executive function [Trails A and B (Reitan, 1971), Verbal Fluency (Lezak, 1995), and Clock Drawing (Sunderland et al., 1989)], language [Boston Naming Short Form (Mack et al., 1992)], constructional praxis from the Consortium to Establish a Registry for AD battery (Morris et al., 1989), memory [Rey Auditory Verbal Learning Test with verbal recall and recognition (Rey, 1941)] recall of constructional praxis for non-verbal memory, and agnosia (Kertesz, 1983). Clinicians used clinical checklists, data from the neuropsychological assessment, neuropsychiatric history, and medical history to formulate consensus diagnoses. Criteria for the following diagnoses were applied: MCI (Petersen et al., 1999; Winblad et al., 2004), age-associated memory impairment (Crook et al., 1987), age-associated cognitive decline (Kral, 1962), mild neurocognitive disorder (American Psychiatric Association, 1994), impairment on the Clinical Dementia Rating Scale (Roberts et al., 2012), and other cognitive disorder (American Psychiatric Association, 1994). Diagnostic and Statistical Manual of Mental Disorders, fourth edition, criteria were used to assess dementia and delirium (American Psychiatric Association, 1994). The Petersen criteria for MCI were used at waves 1 and 2 (Petersen et al., 1999), whereas the Winblad criteria (Winblad et al., 2004) were used at wave 3. For all other categories, the same criteria were used for all three waves and have been published by our group elsewhere (Kumar et al., 2005). Important for this study, clinicians were blind to the presence or lack of diagnosis obtained at waves 1 and 2.

### Dietary measures

Dietary intake was measured using the Commonwealth Scientific and Industrial Research Organization (CSIRO) Food Frequency Questionnaire (FFQ) (Baghurst and Record, 1984), which consists of questions on 215 food items as well as details about cooking methods, serving sizes, comments about eating habits, and the use of dietary supplements. Reported information in conjunction with food composition tables for Australia was used to calculate participants’ daily nutrient intake. This method has been shown to have high repeatability and consistency with other dietary intake measurement techniques and has demonstrated good reliability compared with urinary and serum measures (Rohan et al., 1987; Lazarus et al., 1995; Lassale et al., 2009). In the present study, the daily intake of calcium (Ca), iron (Fe), magnesium (Mg), and potassium (K) were considered. In order to account for the effect of total caloric intake, the raw intake of minerals was normalized to a daily caloric intake of 10 MJ. Effects of calcium, magnesium, and potassium were evaluated per 100 mg increments while the iron effect was investigated in 1 mg increments.

### Covariates

Alcohol intake was assessed using the Alcohol Use Disorders Identification Test (AUDIT) and grouped into none/mild, moderate, harmful/hazardous (Saunders et al., 1993). Present and past smoking status was assessed by self-report. Body mass index (BMI) was computed with the formula weight (kilogram)/height^2^ (square meter), where height and weight were self-reported. Stroke and diabetes were assessed by self-report (yes/no). Hypertension was assessed based on blood pressure measures (systolic >140, diastolic >90) or use of anti-hypertensive medication. Apolipoprotein E e4 allele (APOE*E4) genotype was assessed by TaqMan assays (Applied Biosystems) from DNA extracted from buccal swabs (Cherbuin et al., 2008). Depressive symptomatology was assessed with the Goldberg scale (Goldberg et al., 1988). Physical activity was assessed based on the UK Whitehall II Study assessment and coded into mild, moderate, and vigorous categories (Stafford et al., 1998).

### Statistical analysis

Descriptive analyses were conducted using Chi-square for categorical data and *t*-tests to compare groups on continuous variables. We calculated Cox proportional hazards models to produce a risk measure in multivariate analyses with MCI or MCD as the dichotomous outcomes and intake of minerals as predictors (Model 1) and while controlling for age, sex, education, APOE*E4 genotype, BMI, physical activity, stroke, diabetes, hypertension, depressive symptomatology, alcohol intake, and smoking (Model 2). The time to event variable was time of diet assessment to first MCI or MCD diagnosis at study assessment. Subjects who were assessed and did develop any MCD were censored at the time of their last follow-up. Quadratic effects were estimated for the associations between the minerals and diagnostic categories to investigate non-linear relationships. Sex-mineral interactions and interactions between significant minerals were tested. Significance is reported at α 0.05 and 0.01 levels.

## Results

Table 1 presents the demographic and neuropsychological characteristics of the study groups (Normal, MCI, and MCD) as well as their daily mineral intake. We identified 27 MCI and 52 MCD individuals from the 1406 participants, who did not have a diagnosis at wave 1 and who were included in the study.

**Table 1:** **Demographic, clinical, dietary, and genetic characteristics of normal, MCI and MCD sub-samples**.

	Normal (*n* = 1354)	MCI (*n* = 27)	MCD (*n* = 52)
Females, *n* (%)	700 (51.70)	10 (37.00)	22 (42.30)
Age wave 1, years (SD)	62.54 (1.53)	62.37 (1.80)	62.50 (1.60)
Education years (SD)	14.15 (2.57)	13.06** (1.91)	13.08** (2.21)
Caucasian, *n* (%)	1321 (97.60)	26 (96.30)	51 (98.10)
**MMSE**			
At wave 1 (SD)	29.43 (0.85)	28.41** (1.22)	28.63** (1.12)
At wave 2 (SD)	29.41 (0.86)	28.27** (1.19)	28.45** (1.24)
At wave 3 (SD)	29.32 (0.97)	27.81** (1.65)	27.74 (1.93)
BMI kg/m^2^ (SD)	26.60 (4.99)	27.27 (4.97)	27.64** (6.36)
**ACTIVITY LEVEL, *n* (%)**			
Low	663 (49.00)	12 (44.40)	29 (55.80)
Moderate	516 (38.10)	13 (48.10)	19 (36.50)
High	175 (12.90)	2 (7.40)	4 (7.70)
Diabetes, *n* (%)	72 (5.30)	6** (22.20)	10** (18.30)
Hypertension, *n* (%)	831 (61.90)	16 (61.50)	31 (60.80)
**ALCOHOL, *n* (%)**			
Abstainers	351 (25.90)	11 (30.70)	16 (30.80)
Moderate	917 (67.70)	16 (59.20)	40 (69.30)
Harmful	86 (6.40)	0 (0.00)	0 (0.00)
Caloric intake MJ (SD)	8.54 (2.27)	9.13 (2.62)	8.77 (2.36)
Present and past smokers, *n* (%)	633 (46.8)	12 (44.40)	26 (50.00)
APOE*E4 carrier, *n* (%)	370 (27.30)	5 (18.50)	14 (28.80)
**DIETARY MINERAL INTAKE PER 10 MJ**			
Calcium, g (SD)	1.21 (0.38)	1.26 (0.42)	1.23 (0.37)
Iron, mg (SD)	17.78 (3.96)	17.39 (4.32)	17.35 (3.82)
Magnesium, mg (SD)	434 (78)	422 (82)	427 (78)
Potassium, g (SD)	4.91 (1.00)	5.03 (1.08)	4.89 (1.12)

Differences between clinical and normal groups: significant at *0.05, **0.01.

*MMSE, mini-mental state examination; BMI, body mass index*.

Average mineral intake in this cohort is generally consistent with advice from the National Health and Medical Research Council (NHMRC) of Australia, which recommends a daily intake of 420 mg of magnesium, 1000 mg of calcium, and 8 mg for iron in men and 420 mg of magnesium, 1000 mg of calcium, and 8 mg for iron in women in their 60s. However, iron intake at more than 18 mg in this sample is substantially higher than the recommended value. There is no NHMRC recommendation for potassium but the reported daily average intake is 3800 mg for men and 2800 mg for women and consistent with that found in this study.

Results from the unadjusted multivariate Cox regression analyses predicting transition from normal cognition to MCI and MCD (Model 1) and after controlling for age, sex, education, BMI, physical activity, diabetes, hypertension, depression, smoking, and alcohol intake category (Model 2) are presented in Table 2 and show that intake of magnesium was associated with a decreased risk of MCI while potassium and iron were associated with an increased risk. None of the mineral by mineral interactions or quadratic effects reached significance. However, for MCI significant sex interactions were detected for magnesium and iron.

**Table 2:** **Dietary predictors of transition from normal aging to MCI or MCD**.

Predictors	MCI				MCD			
	Model 1		Model 2		Model 1		Model 2	
	HR (95% CI)	*P*	HR (95% CI)	*P*	HR (95% CI)	*P*	HR (95% CI)	*P*
Calcium	1.02 (0.99–1.01)	0.767	1.01 (0.89–1.15)	0.866	1.05 (0.96–1.14)	0.311	1.05 (0.96–1.14)	0.309
Iron	1.50 (1.07–2.10)	0.019	1.54 (1.03–2.29)	0.034	1.05 (0.95–1.16)	0.309	1.10 (0.87–1.37)	0.418
Magnesium	0.08 (0.02–0.47)	0.005	0.07 (0.01–0.56)	0.013	0.45 (0.21–0.96)	0.039	0.47 (0.22–0.99)	0.046
Potassium	1.09 (1.02–1.17)	0.016	1.09 (1.01–1.17)	0.028	1.05 (0.99–1.11)	0.070	1.05 (0.99–1.10)	0.107
**INTERACTIONS**								
Sex × magnesium	2.87 (1.02–7.97)	0.046	3.41 (0.92–12.65)	0.068				
Sex × iron	0.73 (0.56–0.94)	0.016	0.71 (0.52–0.97)	0.031	0.97 (0.93–0.99)	0.045		

*Model 1: unadjusted; Model 2: adjusted for age, sex, education, body mass index, activity level, diabetes, hypertension, depression, smoking, alcohol intake*.

*MCI, mild cognitive impairment; MCD, mild cognitive disorder; HR, hazard ratio; CI, confidence interval*.

**Figure 2:**
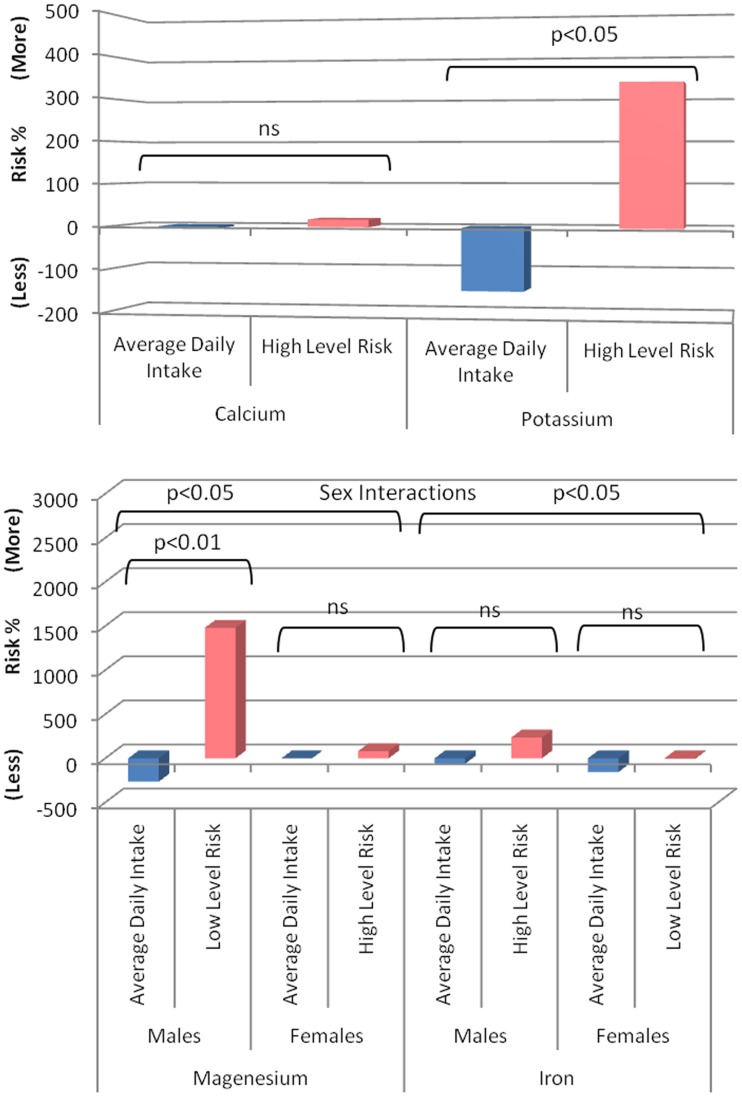
**Risk of developing MCI associated with minerals of interest in cognitively healthy individuals (blue, based on observed group mean intake relative to recommended intake from the NHMRC) compared to the risk associated with the NHMRC upper limit (pink) or were no upper limit is available the observed value in the present sample associated with greatest risk**. A positive value represents a percent increased risk and a negative value a percent decreased risk.

Follow-up analyses demonstrated that higher magnesium intake was associated with a decreased MCI risk in males (HR 0.14, 95% CI 0.03–0.60, *p* = 0.008) but not in females (HR 1.36, 95% CI 0.26–7.08, *p* = 0.714) and suggested (trends) that higher iron intake was associated with a decreased risk of MCI in females (HR 0.81, 95% CI 0.61–1.07, *p* = 0.144) and an increased risk in males (HR 1.08, 95% CI 0.94–1.18, *p* = 0.287). Visual representations of the estimated risk for each mineral relative to NHMRC recommended values and upper limits are presented in Figure 2.

### Additional analyses

To shed light on the sex interactions detected, magnesium and iron intake per 10 MJ was contrasted between males and females. For magnesium, male (mean 415 mg, SD 76) was lower than female (mean 451 mg, SD 77) intake in cognitively intact individuals (*t* = −8.55, *p* > 0.001) but even more so in MCI participants (Males: mean 403 mg, SD 81; Females: mean 468 g, SD 55; *t* = −2.25, *p* < 0.05). For iron, male (mean 17.11 mg, SD 3.80) was lower than female (mean 18.38 mg, SD 4.00) intake in cognitively intact individuals (*t* = −6.02, *p* > 0.001) but not in MCI participants (Males: mean 17.82, SD 4.99; Females: mean 16.66 SD 2.96; *t* = 0.666, ns).

In addition, as in the present analyses mineral intake was considered as a density per 10 MJ to provide an easily understandable metric and since this approach can potentially confound the effects of the minerals investigated with that of caloric intake, further analyses were conducted to exclude this possibility. Firstly, analyses were repeated using caloric intake as an additional covariate and secondly using the approach suggested by Willett et al. (1997) the variance in caloric intake shared with mineral intake measures was partialed out. This was done by regressing the mineral intake on caloric intake and saving the residuals. For each mineral the mean cohort intake per 10 MJ was added to the residuals. This does not affect the statistical effects but “standardizes” nutrient intake to an average typical for a known caloric intake. In both sets of analyses (Supplementary Tables 1 and 2), results followed a similar and consistent pattern suggesting caloric intake is not the underlying factor that produced the present results.

## Discussion

The most significant findings emerging from this study were (1) that dietary intake of minerals implicated in AD pathology and risk of developing AD (iron, magnesium, potassium) were found to be associated with risk of progression from normal aging to MCI and MCD and (2) that for magnesium and iron these associations differed between sexes.

As predicted, higher intake of magnesium was associated with a decreased risk of MCI/MCD. This is consistent with previous research but nevertheless interesting since here it relates to mild cognitive disorders thus suggesting a continuum of effects spanning cognitive decline in aging, MCDs, and dementia. However, this effect appeared to be only present in males who had overall a lower intake of magnesium than females but even more so in the MCI/MCD groups. These findings are particularly interesting in light of recent research in animal models of AD. Li et al. (2013) found that rats whose diet was complemented with magnesium (in drinking water) suffered less synaptic loss in the hippocampus, developed less amyloid plaques, and demonstrated preserved learning and memory capacities compared to controls. The authors also noted that long-term magnesium supplementation increased CSF concentration by only 15% while total brain concentration increased by 30%. This suggests that small increases in magnesium intake may lead to substantial brain changes particularly since additional investigation showed that increasing intra-cellular magnesium by 15% led to a 50% increase in synaptic density. These findings are also consistent with a post-mortem study, which found that the magnesium content in the hippocampus of AD patients was reduced by 18% compared to controls (Andrasi et al., 2005).

Surprisingly, no effect of calcium and an opposite effect of potassium were found. In relation to potassium, it may be that the risk associated with its intake is not linear and that it is a protective factor earlier in the disease process, as shown by Ozawa et al. (2012) over a 17-year follow-up – likely through positive effects on cardio-vascular function – and that it becomes a risk factor closer to clinical diagnosis. This hypothesis is consistent with recent findings showing higher potassium concentrations in brains of individuals with AD compared to controls (Vitvitsky et al., 2012). Similarly, calcium may be protective earlier in life but may be ineffective in altering existing cardio-vascular pathology and thus may not alter cerebral health and function in older individuals.

An effect of dietary iron was detected suggesting the presence of an overall increased risk in association with higher iron intake. In addition, a significant sex interaction was present and although follow-up analyses only provided trends, probably due to smaller cell sizes, they hinted at a protective effect of iron in females and an increased risk in males. The sex difference does not appear to be due to variations in dietary concentrations as females had higher iron intakes than males. This gender effect may therefore be due to physiological differences or to a combination of factors such as overall diet, physical fitness, and general health.

While an investigation or detailed discussion of specific foods associated with mineral intake is beyond the scope of this study, it is useful to note that the foods with the highest content in magnesium include green vegetables, nuts and seeds, and unrefined grains all of which have been shown to have health benefits and specifically in relation to their cardio-vascular and anti-oxidant effects as well as their low GI nature (Ness and Powles, 1997; Kang et al., 2005; Dauchet et al., 2006; Brand-Miller and Buyken, 2012; Martinez-Lapiscina et al., 2013). Consequently, it is possible that the associations detected between magnesium and risk of cognitive decline are at least in part attributable to other qualities of foods that contain it, particularly since diets rich in vegetables and fish have been shown to be associated with a lower risk of dementia (Solfrizzi et al., 2011; Otaegui-Arrazola et al., 2014). Magnesium is also necessary for efficient calcium uptake. Potassium-rich food include pumpkin, potatoes, bananas, oily fish, yogurt, and dried fruit, which are generally thought to be healthy foods, therefore the negative effect detected in the present study may relate to the way in which some of these foods are prepared (e.g., chips). This question requires further investigation in future research. Finally, foods high in iron include red meat, oily fish, wholegrain cereal, and dark green vegetables. Gender differences associated with iron intake may be due to physiological differences between men and women but could also be driven by different dietary patterns, which can have opposite effects. For example, low iron levels in women are associated with a higher risk of depression which is a risk factor for dementia. In contrast, high intake of red meat, which is rich in iron, is associated with a higher risk of cardio-vascular disease.

Although, based on current evidence it is reasonable to hypothesize that mineral intake may lead to cognitive decline, reverse causation is also possible whereby those in the pre-clinical stages of dementia alter their diet. This explanation seems less likely here as all individuals included in the study were in their early 60s, cognitively healthy at baseline, and because the outcome measure was not dementia itself but mild cognitive disorders.

This study had a number of limitations but also significant strengths. The cohort surveyed may not generalize to other populations and due to the correlational nature of this research our findings may be accounted for by other factors, which covary with dietary intake, including other nutrients that co-occur in foods rich in the minerals examined. To decrease this possibility, we took care in controlling for important covariates and particularly BMI and total caloric intake. Since dietary intake was assessed by self-report mineral intake measures are likely to be somewhat less reliable than those of more objective methods although a validated FFQ was used. However, this should have led to decreased statistical power, which makes the strong findings demonstrated in this research all the more notable. It should also be noted that intake of nutrients is not equivalent to the dose absorbed as different individuals have different absorption rates under different conditions. Unfortunately, blood measures were not available to explore how dietary intake relates to plasma concentrations and this question should be further investigated in future studies. Despite these limitations, this study’s strengths included a large cohort of individuals selected randomly from the electoral roll, followed longitudinally and assessed for cognitive impairment using a clinical interview and validated criteria.

In summary, this investigation provides new evidence linking increased magnesium intake with decreased risk of cognitive impairment. Importantly, these findings were detected earlier in the disease process than previous studies, which focused on dementia and therefore are more consistent with exposure to longer-term diet quality rather than change in dietary intake shortly before the onset of dementia. This study also produced unexpected results, which may be due to the heterogeneous nature of the MCI syndrome, regional difference in diet quality, or non-linear effect along the disease continuum, which will need to be clarified in future research.

## Conflict of Interest Statement

The authors declare that the research was conducted in the absence of any commercial or financial relationships that could be construed as a potential conflict of interest.

## Supplementary Material

The Supplementary Material for this article can be found online at http://www.frontiersin.org/Journal/10.3389/fnagi.2014.00004/abstract
