# Supplementary material for: Dietary Mineral Intake and Risk of Mild Cognitive Impairment: The PATH through Life Project
Source: Front Aging Neurosci. 2014 Feb 4;6:4. doi: 10.3389/fnagi.2014.00004 (PMC3912433; doi:10.3389/fnagi.2014.00004)
Supplement: Supplementary file 1 [file 72426_Cherbuin_DataSheet1.PDF]

Supplementary Table 1. Dietary predictors (per 10MJ) of transition from normal aging to MCI or MCD after controlling for caloric intake.

Predictors	MCI				MCD			
	Model 1		Model 2		Model 1		Model 2	
	HR (95% CI)	P	HR (95% CI)	P	HR (95% CI)	P	HR (95% CI)	P
Calcium	1.00 (0.99 - 1.01)	0.769	1.00 (0.99 - 1.01)	0.960	1.00 (1.00 -1.01)	0.311	1.04 (0.95 -1.14)	0.362
Iron	1.50 (1.07 - 2.10)	0.019	1.56 (1.06 - 2.37)	0.026	1.05 (1.00 -1.16)	0.309	1.01 (0.92 -1.11)	0.810
Magnesium	0.08 (0.02 - 0.57)	0.005	0.07 (0.01 - 0.62)	0.017	0.45 (0.21- 0.96)	0.040	0.48 (0.23- 1.02)	0.057
Potassium	1.09 (1.02 – 1.18)	0.016	1.14 (1.02 – 1.20)	0.027	1.05 (0.99- 1.11)	0.070	1.05 (0.99- 1.11)	0.089
Interactions								
Sex * Magnesium	1.84 (1.02– 7.95)	0.047	3.42 (0.89 – 13.05)	0.072				
Sex * iron	0.73 (0.56– 0.974)	0.016	0.69 (0.51– 0.95)	0.025	0.97 (0.93-0.99)	0.045		

Model 1: unadjusted; Model 2: adjusted for age, sex, education, BMI, activity level, diabetes, hypertension, depression, smoking, alcohol and caloric intake

MCI: Mild Cognitive Impairment; MCD: Mild Cognitive Disorder; HR: hazard ratio

Supplementary Table 2. “Standardised” dietary predictors of transition from normal aging to MCI or MCD.

Predictors	MCI				MCD			
	Model 1		Model 2		Model 1		Model 2	
	HR (95% CI)	P	HR (95% CI)	P	HR (95% CI)	P	HR (95% CI)	P
Calcium	1.04 (0.91 - 1.19)	0.565	0.99 (0.85 - 1.15)	0.882	1.06 (0.96 -1.17)	0.243	1.05 (1.00 -1.01)	0.289
Iron	1.15 (0.98 – 1.35)	0.098	1.79 (1.15 - 2.79)	0.010	1.06 (0.95 -1.18)	0.318	1.14 (0.87 -1.48)	0.302
Magnesium	0.04 (0.01 - 0.42)	0.007	0.03 (0.01 - 0.35)	0.006	0.32 (0.13- 0.80)	0.015	0.37 (0.15- 0.91)	0.030
Potassium	1.15 (1.05 – 1.26)	0.002	1.14 (1.04 – 1.26)	0.035	1.07 (0.93- 0.99)	0.034	1.07 (0.99- 1.14)	0.053
Interactions								
Sex * Magnesium	3.19 (0.79 – 12.85)	0.102	5.35 (1.12 – 25.55)	0.035				
Sex * iron	0.90 (0.64– 0.968)	0.004	0.63 (0.4– 0.90)	0.012	0.97 (0.93-0.99)	0.033		

Model 1: unadjusted; Model 2: adjusted for age, sex, education, BMI, activity level, diabetes, hypertension, depression, smoking, alcohol and caloric intake

MCI: Mild Cognitive Impairment; MCD: Mild Cognitive Disorder; HR: hazard ratio
